# The effect of indomethacin, myeloperoxidase, and certain steroid hormones on bactericidal activity: an ex vivo and in vivo experimental study

**DOI:** 10.1186/1476-0711-13-27

**Published:** 2014-07-07

**Authors:** Júlia Stark, Zsuzsanna Varga, Ágoston Ghidán, Péter Vajdovich, Dezső Szombath, István Marczell, Szabolcs Várbíró, Elek Dinya, Tibor Magyar, Zsolt Tulassay, Béla Székács, Károly Nagy, Károly Rácz, Gábor Békési

**Affiliations:** 1 Faculty of Medicine, 2nd Department of Internal Medicine, Semmelweis University, 46. Szentkiralyi u., H-1088 Budapest, Hungary; 2 Hungarian Academy of Sciences, Institute for Veterinary Medical Research, 21. Hungaria krt., H-1143 Budapest, Hungary; 3 Faculty of Medicine, Institute of Medical Microbiology, Semmelweis University, 4. Nagyvarad ter, H-1089 Budapest, Hungary; 4 Department and Clinic of Internal Medicine, Szent Istvan University, Faculty of Veterinary Science, 2. Istvan u., H-1078 Budapest, Hungary; 5 Faculty of Medicine, Institute of Patophysiology, Semmelweis University, 4. Nagyvarad ter, H-1089 Budapest, Hungary; 6 Faculty of Medicine, 2nd Department of Obstetrics and Gynecology, Semmelweis University, 78/a Ulloi ut, H-1082 Budapest, Hungary; 7 Faculty of Health and Public Services, Institute of Health Informatics Development and Further Training, Semmelweis University, 78/b Ulloi ut, H-1082 Budapest, Hungary

**Keywords:** Bacterial killing activity, Infection, Myeloperoxidase, Indomethacin, Testosterone, Estradiol, Hydrocortisone

## Abstract

**Background:**

The role of myeloperoxidase (MPO) is essential in the killing of phagocytosed bacteria. Certain steroid hormones increase MPO plasma concentration. Our aim was to test the effect of MPO, its inhibitor indomethacin, and certain steroid hormones on bactericidal activity.

**Methods:**

Human polymorphonuclear leukocytes (PMN) were incubated with opsonised *Escherichia coli* and either MPO, indomethacin, estradiol, or hydrocortisone. Intracellular killing capacity was evaluated with UV microscopy after treatment with fluorescent dye. Next, an *in vivo* experiment was performed with nine groups of rats: in the first phase of the study indomethacin treatment and *Pasteurella multocida* infection (Ii), indomethacin treatment without infection (I0), untreated control with infection (Mi) and untreated control without infection (M0); in the second phase of the study rats with infection and testosterone treatment (NT), castration, infection and testosterone treatment (CT), castration, infection and estradiol treatment (CE), non-castrated infected control (N0), and castrated infected control (C0). After treatment bacteria were reisolated from the liver and heart blood on agar plates, and laboratory parameters were analyzed. For the comparison of laboratory results ANOVA or Kruskal-Wallis test and LSD post hoc test was used.

**Results:**

Indomethacin did not have a remarkable effect on the bacterial killing of PMNs, while the other compounds increased bacterial killing to various degrees. In the animal model indomethacin and infection caused a poor clinical state, a great number of reisolated bacteria, elevated white blood cell (WBC) count, decreased C-reactive protein (CRP) and serum albumin levels. Testosterone treatment resulted in less bacterial colony numbers in group NT, but not in group CT compared to respective controls (N0, C0). Estradiol treatment (CE) decreased colony numbers compared to control (C0). Hormone administration resulted in lower WBC counts, and in group CE, a decreased CRP.

**Conclusions:**

MPO, estradiol, and hydrocortisone improve bacterial killing activity of PMNs. Indomethacin treatment and castration weaken immune responses and clinical state of infected rats, while testosterone and estradiol have a beneficial effect.

## Background

Free radicals are very reactive compounds with one or more unpaired electrons on their outer orbitals. They are of great importance in the bacterial killing role of macrophages. The most important reactive oxygen radical is the superoxide anion with limited reactivity, but it can be converted into other more reactive radicals. Its main source is NADPH, which is oxidased by NADPH oxidase (NOX). Superoxide anion can be converted into hydrogen peroxide (H_2_O_2_) by superoxide dismutase (SOD), and the produced H_2_O_2_ is used in the oxidation of chloride ions to hypochlorous acid (HOCl) with the catalyzation of myeloperoxidase (MPO). HOCl deploys bactericidal activity on the bacteria ingested in the phagosome. Additionally, chloramines generated by HOCl reacting with phagosomal proteins also contribute to bacterial killing, but the full consequences of this are not yet clear [1,2].

However, under certain circumstances an excess of these oxidizing species can overwhelm local antioxidant defence and lead to oxidative stress (OS) and tissue injury that are implicated in the pathogenesis of diseases like atherosclerosis, emphysema, acute respiratory distress syndrome, reperfusion injury, malignancy, diabetes mellitus, and Alzheimer’s disease [2].

The tissues are protected against free radicals by antioxidant enzymes (such as SOD, catalase, glutathione reductase, α_1_-antitripsin), peptides with thiol groups (glutathione, thioredoxin family) and nutritional antioxidants (vitamin C and E, carotenoids, trace elements) [2,3]. Certain steroid hormones also play a role in antioxidant defence through various mechanisms. In our previous work we demonstrated that cortisol, 17-ß-oestradiol (E_2_), progesterone, and testosterone decreased the superoxide release from human PMNs, while aldosterone and cortexolone, the precursor of cortisol did not have such property [4].

In the literature the most extensively studied steroid hormone is E_2_. It enhances the cellular anti-oxidative defence molecules, reduces the production of reactive oxygen species (ROS), activates endothelial, inducible and neuronal nitric oxide synthase, and neutralizes the excess ROS in various cell types [5].

The mechanism of antioxidant action of hydrocortisone is scantly studied in the literature. It is mainly referred to as the main biomarker of stress, which leads to oxidative changes [6]. However, a correlation between plasma hydrocortisone and Cu/Zn SOD activity has been recorded in rhesus monkeys, and both have been found to have a similar diurnal rhythm [7]. Other glucocorticoids have both glucocorticoid receptor mediated genomic and a rapid non-genomic antioxidant effect [8,9].

The antioxidant effect of testosterone is ambiguous in the literature. In one study it showed a receptor-mediated antioxidant effect in vitro on cerebellar granule cells [10], but according to others it caused a decrease in the activity of antioxidant enzymes and led to lipid peroxidation in the prostate and testis of rodents [11,12].

MPO has also been reported to have antioxidant capacity despite its known free radical producing activity. It contributes to the termination of free radical production in PMNs [13,14], and the incubation of human PMNs with MPO and NaClO, the end-product of the MPO catalyzed peroxidation, yielded a decrease in superoxide release, probably by inhibiting NOX [14,15]. In fact, it has been found, that MPO release and activity in PMNs can be enhanced by certain steroids, like E_2_, testosterone, and prednisolone. Thus, the antioxidant effect of these steroid hormones might be carried out at least in part by the elevation of MPO activity and release [14-16]. On the other hand, the MPO inhibitor indomethacin, a non-steroidal anti-inflammatory drug, a nonselective cyclooxygenase inhibitor increases free radical production in PMNs [14,17]. This could mean a beneficial effect in the bactericidal activity of PMNs.

In our present studies we aimed to evaluate the effect of indomethacin on *ex vivo* and *in vivo* bactericidal function. We also tested MPO, E_2_, and hydrocortisone on antibacterial capability of human PMNs, and the effect of indomethacin, testosterone, and E_2_ on an *in vivo* rat model. Our ultimate aim was to evaluate if indomethacin can be useful as an adjuvant therapy in septic patients. Since the two experimental models are different, and sex steroids have various effects on many participants of the immune system besides PMNs, the comparison of the results of the two experiments needs caution.

## Methods

### *Ex vivo* effect of indomethacin, MPO, estradiol, and hydrocortisone on the bactericidal activity of human isolated PMNs

#### Isolation of PMNs

Venous blood was drawn from 6 male and 5 female donors, aged 23–40 years. All volunteers were non-smokers, took no medications, did not suffer from any known disease and consented to participation. The blood was taken into EDTA tubes between 8–9 a.m. after an overnight fasting. PMNs were separated within 2 hours of blood drawing with the following method. The blood was applied to Histopaque (Sigma, 1077–1) in layers for the sedimentation of red blood cells and put aside for an hour. Then it was transferred to 63% and 72% Percoll (Sigma, P-1644) and centrifuged for 25 min at a rate of 300 × g at 20°C. Granulocytes thus separated were buffer-washed twice and centrifuged with 220 × g. Cells aggregated at the bottom of the tube were re-suspended in a few milliliters of Hank’s buffer salt solution (Sigma, H-8264), then counted using Turk’s solution. The cell concentration of the suspension was adjusted to 5 million cells/ml.

#### *E. coli opsonisation*

*Escherichia coli* O111:K58 strain was used. The bacterial strain was cultivated on blood agar plates overnight at 37°C. A loopful of bacteria was suspended in 200 μl of Hank’s buffer. After adding 4 drops of antibody to the suspension it was incubated for 30 min at 37°C. The antibody was specifically produced for *E. coli* O111:K58 strain by the National Centre of Epidemiology, Budapest, Hungary.

#### Intracellular bacterial killing

400 μl (2×10^6^) of 5×10^6^/ml PMNs were incubated for 2 hours at 37°C in a water bath either with 0.01 mg/ml indomethacin (Sigma, I7378-5G), 71 ng/ml MPO (Merck, 475911), 10^−8^ mol 17ß-oestradiol (Sigma, E8875) or 10^−8^ mol hydrocortisone (Sigma, H4001). Control cells were incubated without any treatment. After incubation, PMNs were mixed with 2×10^7^ opsonised *E. coli* cells, and the compounds were kept in the water bath for further 30 min. After this, 1–1 ml of ice-cold Hank’s buffer was added to each compound, and they were centrifuged for 7 min at 6000 rpms. After having removed the aliquots, the pellets were dyed with 200 μl (1.44 ml/100 ml Hank’s buffer) acridine orange (Sigma, A6014-10G) for one minute. Then again 1 ml cold Hank’s buffer was added and the mixtures were centrifuged for 5 min at 10,000 rpms. The aliquots were removed and bacterial killing was examined in the pellets. Acridine orange stains only dead bacteria, which fluoresce in orange when excited by ultraviolet light. The bacterial killing effect was evaluated visually under UV microscope (Leica, DMRB DIC). Several fields of vision were examined for each sample in order to find the true amount of dead bacteria. To express intracellular killing effect of the various drugs a scoring system from 0–5 was set up, where 0 indicates no bacterial killing and 5 indicates a hypothetical maximal killing. The results of bacterial killing were also expressed as percentages compared to the control, which is considered 100%. From the results of parallel measurements an average was calculated.

### Effect of indomethacin, testosterone, and estradiol on the bactericidal activity *in vivo* in rats

The *in vivo* experiments were carried out in two phases. SPF adult male Wistar rats {Crl.(Wi)Br.} weighing 120–170 grams were used for both experiments. In the first phase animals were kept in groups of eight, had access to conventional rat food (Ssniff R-Z, Spezialdiaten GmbH, Soest, Germany) and fresh drinking water ad libitum. The rats were given the following treatments through a feeding tube for five days, once daily: groups M0 and Mi: 0.8 ml of 1% methylcellulose solution; groups I0 and Ii: 0.8 mg indomethacin [0.8 ml of 1 mg/ml indomethacin (Sigma, 865500-5G) dissolved in 1% methylcellulose]. In the names of the groups, the uppercase letter refers to the treatment (M = methylcellulose, I = indomethacin), “0” means no infection and “i” refers to infection.

On the 3rd day of treatment rats in groups Mi and Ii were infected with *Pasteurella multocida ssp. septica.* Prior to infection, *Pasteurella multocida ssp. septica* (isolate P964) was stored in 1 ml of skimmed milk at −70°C. The isolate was streaked onto Columbia agar plate (Merck, Darmstadt, Germany) containing 5% (v/v) defibrinated sheep's blood and cultivated at 37°C for 24 hours. Single colonies were passed and cultivated again at 37°C for 24 hours. Bacterium culture was washed with 2 ml saline. Rats were infected with a subcutaneous injection of 0.2 ml of 6×10^8^ colony forming units (CFU)/ml bacteria diluted in physiological saline (1.2×10^8^ germs/animal). Uninfected animals got 0.2 ml saline on the same route.

On the 5th day of treatment animals were anaesthetized with diethyl ether and exsanguinated through the abdominal aorta. Blood was collected for laboratory analysis of inflammatory markers and hormone levels. Blood count, serum proteins, CRP, serum testosterone and estradiol were determined with well established automated laboratory methods used in clinical practice at the Szent Istvan University, Faculty of Veterinary Science, Department and Clinic of Internal Medicine, Budapest.

Isolation of the infective agent was attempted from each liver and heart blood. A loopful of sample was streaked onto Columbia blood agar (CBA) and plates were incubated at 37°C for 24 hours. The number of CFUs was evaluated and isolates were classified as “none”, “1-10 colonies”, “dense, but separate” and “confluent” colony patterns according to the density of the culture. The uninfected groups were negative for culturing while the majority of infected animals gave positive result with variable density. Microbiological examinations were carried out at the Institute for Veterinary Medical Research of the Hungarian Academy of Sciences, Budapest.

In the second *in vivo* phase 30 rats were used and all of the animals were infected and the effects of hormone treatments were examined. Animals were kept as described previously. Five groups of 6 were created, and 18 rats were castrated. After a 10-day recovery period the following treatments were given for seven days, twice daily (8:30 a.m., 6 p.m.) in a subcutaneous injection: group N0: 0.35 ml propylene glycol, not castrated; group NT: 0.35 ml of 800 μg/ml testosterone dissolved in propylene glycol, not castrated; group C0: 0.35 ml propylene glycol, castrated; group CT: 0.35 ml of 800 μg/ml testosterone dissolved in propylene glycol, castrated; group CE: 0.35 ml of 20 μg/ml 17-ß-estradiol dissolved in propylene glycol, castrated. In the names of the groups “N” and “C” mean not castrated and castrated, respectively; “0”, “T” and “E” refer to no treatment, testosterone and estradiol treatment, respectively.

On the 4th day of treatment all animals were infected with a subcutaneous injection of 0.2 ml of 2.4×10^9^ CFU/ml *Pasteurella multocida ssp. septica* diluted in physiological saline, as described above (4.8×10^8^ germs/animal). On the 7th day of treatment animals were anaesthetized and exsanguinated, laboratory and microbiological examinations were carried out as in the previous experiment.

Both experiments were approved by the university ethical committee.

### Statistical analysis

The Statistica software was used for all analyses. For the comparison of laboratory results of rats ANOVA or Kruskal-Wallis test and LSD post hoc test was used. *P* < 0.05 was considered statistically significant.

## Results

### *Ex vivo* effect of indomethacin, MPO, estradiol, and hydrocortisone on the bactericidal activity of human isolated PMNs

**Figure 1:**
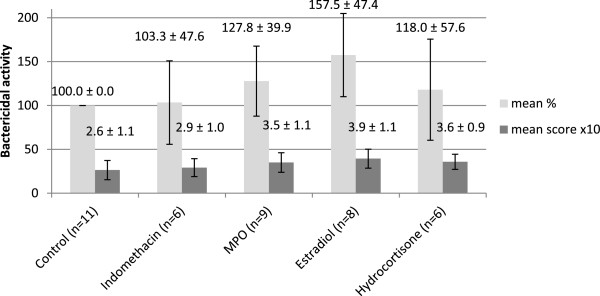
**The bactericidal activity of PMNs after indomethacin, myeloperoxidase (MPO), estradiol, and hydrocortisone treatment.** Results are expressed as percentage of control (mean ± SD) and as scores (mean ± SD). For better visibility the scores were multiplied by 10 on the diagram. The error bars show standard deviation.

The *ex vivo* effect of indomethacin, MPO, estradiol and hydrocortisone on bacterial killing are shown in Figure 1. Due to technical problems not all experiments gave valuable results, thus the number of donors (n) is variable among treatment groups. Indomethacin did not have a remarkable effect in either direction, using either scale. The three other compounds seem to increase bacterial killing of PMNs to various degrees: estradiol is the most efficient, while hydrocortisone has the least activity.

### Effect of indomethacin, testosterone, and estradiol on the bactericidal activity *in vivo* in rats

**Figure 2:**
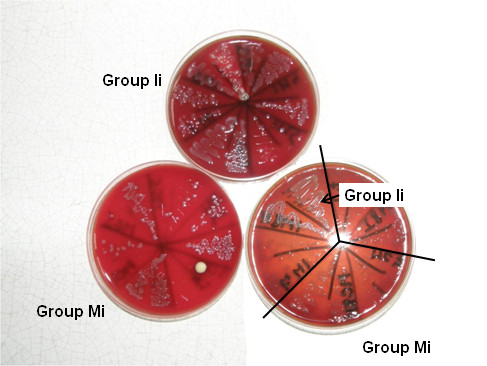
**Reisolation of *P. multocida *from infected rats in the first phase of the animal study.** Members of group Ii received indomethacin treatment and were infected, members of group Mi received no treatment and were infected.

In the first phase of our *in vivo* studies 1–1 rats were wasted in the infected groups (Mi, Ii), and 2 in the uninfected group treated with indomethacin (group I0) on the 4th day of treatment, i.e. the day after the infection. Animals in groups I0 and Ii (treated with indomethacin, without and with infection) were weak and faint. Ascites was found in two rats in group I0 and one in group Ii. After the reisolation of *P. multocida* from liver and cardiac blood of infected animals, colony patterns were evaluated (Table 1, Figure 2). The greatest number of bacteria could be reisolated from the animals in group Ii, with confluent colonies in 11 samples, *vs* 3 samples in group Mi. In group Ii none of the samples gave a negative reisolation, however, in three samples in group Mi no colonies were reisolated. The laboratory findings (Table 1) showed that WBC count was significantly higher in group Ii than in groups I0 and Mi, with higher neutrophil and lower lymphocyte ratios in both indomethacin-treated groups (I0, Ii). In both groups I0 and Ii anaemia was found, with lower red blood cell (RBC), haemoglobin (HGB) and haematocrit (HCT) levels compared to the other groups. Albumin levels were also lower in groups I0 and Ii. CRP was increased in group Mi compared to uninfected groups, however, in group Ii CRP decreased. Laboratory analysis could not be carried out in some samples (2 in group M0, 1 in group I0, 1 in group Mi) due to clotting or insufficient amount of blood.

**Table 1:** Results of the first phase of the animal study

	**1. M0**	**2. I0**	**3. Mi**		**4. Ii**		***p < 0.05***
Treatment	None (methylcellulose)	Indomethacin	None (methylcellulose)		Indomethacin		
Infection	No	No	Yes		Yes		
Colony patterns:			Heart blood	Liver	Heart blood	Liver	
None			2	1	0	0	
1-10 colonies			3	2	2	1	
Dense, but separate			1	4	1	1	
Confluent			2	1	5	6	
Sample number for lab tests	6	5	6		7		
WBC (×10^9^/l)	7.72 ± 2.06	5.09 ± 3.53	5.95 ± 2.61		9.28 ± 1.87		4 vs 2,3
Neutrophil (%)	18.00 ± 4.53	47.40 ± 26.10	22.83 ± 5.64		30.83 ± 9.30		ns
Lymphocyte (%)	80.00 ± 4.36	49.00 ± 28.10	73.67 ± 6.47		64.50 ± 9.22		ns
RBC (×10^12^/l)	5.94 ± 0.68	3.86 ± 1.52	5.66 ± 0.39		3.66 ± 1.09		4 vs 1
HGB (g/dl)	110.00 ± 9.00	73.40 ± 29.20	105.67 ± 6.83		68.50 ± 18.22		4 vs 1
HCT (l/l)	0.39 ± 0.03	0.26 ± 0.11	0.38 ± 0.03		0.25 ± 0.07		4 vs 1
Total protein (g/l)	55.65 ± 2.49	52.20 ± 4.65	59.22 ± 3.00		42.00 ± 7.86		4 vs 3
Albumin (g/l)	27.92 ± 0.98	22.88 ± 3.13	26.07 ± 0.65		19.80 ± 3.65		4 vs 1
Globulin (g/l)	27.73 ± 1.63	29.32 ± 2.79	33.15 ± 2.63		22.20 ± 4.30		3 vs 1,2,4; 4 vs 1,2
CRP (mg/dl)	300.00 ± 25.20	306.60 ± 32.90	411.67 ± 55.98		261.71 ± 72.99		3 vs 1,2,4

The number of different *P. multocida* colony shapes and laboratory results (means ± SD). The last column shows the groups where significant differences were found. ns: not significant.

**Figure 3:**
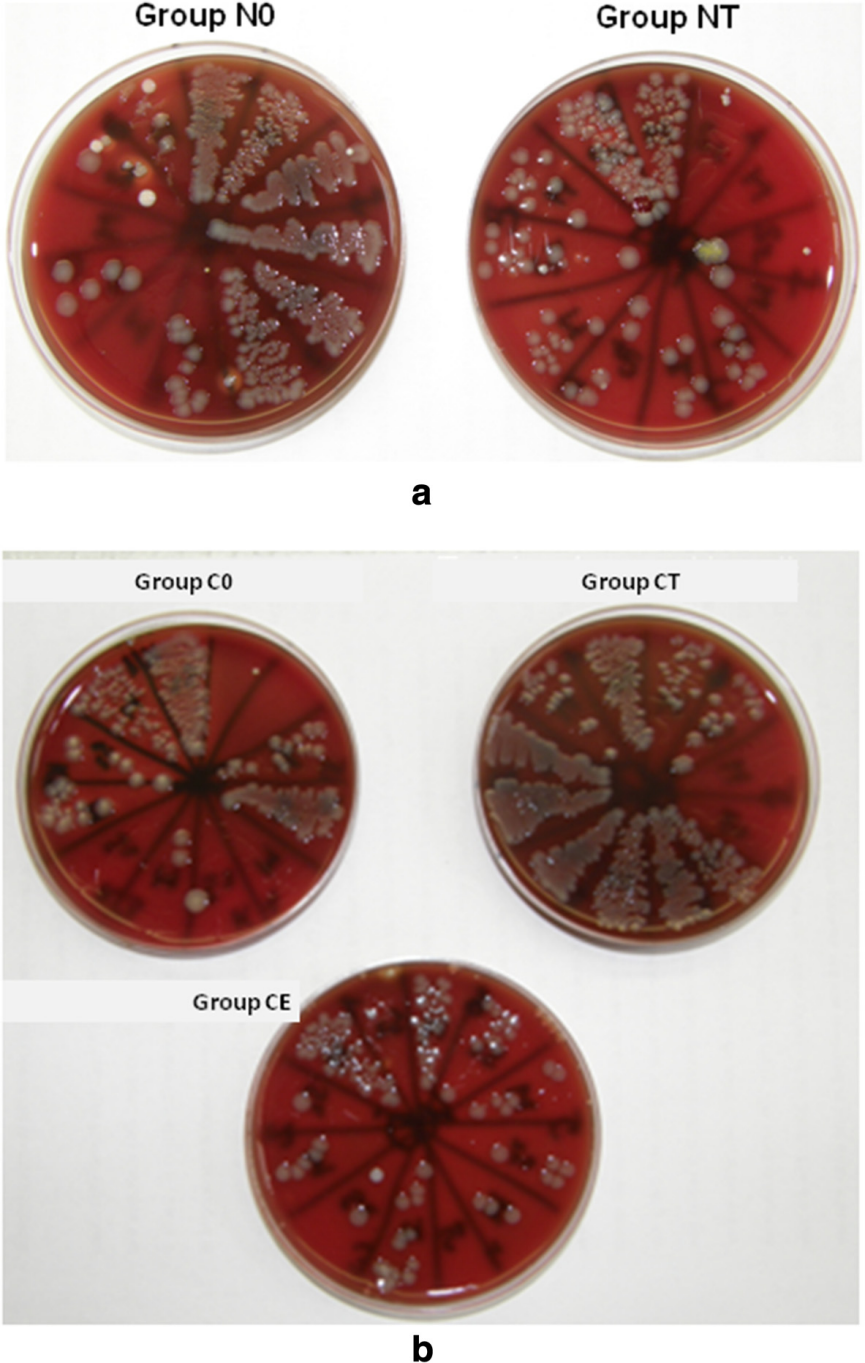
**Reisolation of *P. multocida *from infected rats in the second phase of the animal study. a)** Members of group N0 received no treatment besides infection, group NT received testosterone treatment. **b)** Members of group C0 were castrated, but did not receive treatment besides infection, members of groups CT and CE were castrated and received either testosterone or estradiol treatment, respectively.

In the second phase of the *in vivo* experiments, during the 10 days of recovery after castration, weight gain of castrated animals was less than that of the not castrated ones, otherwise they were recovering fine. One animal in group C0 was wasted before the infection without any clinical antecedent. The section did not reveal any cause, and liver, heart blood and lung tissue cultures gave negative results. After the induction of infection all animals were depressed and faint, especially in group C0. The results of the reisolation of *P. multocida* are shown in Table 2 and Figure 3a-b. In group NT fewer bacteria could be cultured compared to the untreated (N0) group. In group CT more bacteria were reisolated than in group NT and C0. Group CE had similar results as group NT.

**Table 2:** Results of the second phase of the animal study

	**1. N0**		**2. NT**		**3. C0**		**4. CT**		**5. CE**		***p < 0.05***
Castrated	No		No		Yes		Yes		Yes		
Treatment	None		Testosterone		None		Testosterone		Estradiol		
Infection	Yes		Yes		Yes		Yes		Yes		
Colony patterns:	Heart blood	Liver	Heart blood	Liver	Heart blood	Liver	Heart blood	Liver	Heart blood	Liver	
None	0	2	1	1	1	1	1	1	1	0	
1-10 colonies	2	1	4	3	2	1	0	0	4	3	
Dense, but separate	2	2	0	2	0	2	1	2	1	3	
Confluent	2	1	1	0	2	1	4	3	0	0	
Sample number for lab tests	6		6		5		6		5		
WBC (×10^9^/l)	10.70 ± 4.18		5.97 ± 3.22		8.26 ± 4.62		3.61 ± 2.14		2.03 ± 1.05		1 vs 4,5
Neutrophil (%)	50.02 ± 21.82		54.30 ± 26.95		54.12 ± 19.20		38.40 ± 26.29		53.15 ± 21.23		ns
Lymphocyte (%)	45.20 ± 22.89		37.46 ± 28.09		39.56 ± 16.32		58.22 ± 23.98		41.15 ± 22.52		ns
RBC (×10^12^/l)	6.63 ± 0.32		6.44 ± 0.45		5.99 ± 0.47		5.41 ± 0.86		5.95 ± 0.21		4 vs 1,2
HGB (g/dl)	135.40 ± 4.67		132.40 ± 7.16		120.60 ± 12.46		111.40 ± 16.68		120.75 ± 4.65		4 vs 1,2
HCT (l/l)	0.41 ± 0.02		0.40 ± 0.03		0.36 ± 0.03		0.32 ± 0.05		0.35 ± 0.02		4 vs 1,2; 5 vs 1
PLT (×10^9^/l)	718.80 ± 298.45		865.40 ± 212.51		875.60 ± 170.04		696.60 ± 127.91		440.75 ± 198.01		5 vs 2,3
Total protein (g/l)	47.32 ± 2.02		49.53 ± 2.88		51.00 ± 4.45		46.83 ± 4.24		49.76 ± 3.71		ns
Albumin (g/l)	16.68 ± 1.17		17.55 ± 1.18		18.30 ± 1.59		17.03 ± 2.14		18.76 ± 1.50		ns
Globulin (g/l)	30.63 ± 2.67		31.98 ± 2.18		32.70 ± 4.67		29.80 ± 3.03		31.00 ± 2.53		ns
CRP (mg/dl)	375.30 ± 64.54		371.58 ± 33.56		363.30 ± 23.90		329.42 ± 52.21		149.60 ± 131.71		5 vs 1,2,3,4
Testosterone (nmol/l)	21.62 ± 16.08		54.87 ± 1.55		0.87 ± 0.31		41.82 ± 12.10		0.68 ± 0.00		1 vs 2,3,4,5; 2 vs 3,5; 4 vs 3,5
Estradiol (pg/ml)	113.44 ± 30.62		nd		151.75 ± 13.12		nd		867.40 ± 558.15		5 vs 1,3

The number of different *P. multocida* colony shapes and laboratory results (means ± SD). The last column shows the groups where significant differences were found. ns: not significant, nd: not determined.

Laboratory findings of this phase of the study (Table 2) showed that WBC count was lower in group NT than in group N0, and in groups CT and CE than in group C0. CRP was lowest in group CE, and also group CT had a lower CRP than group C0, however, this did not reach significance. One blood sample in group CE was not sufficient for analysis.

## Discussion

In our *ex vivo* experiments we found that the MPO inhibitor indomethacin did not improve the bactericidal activity of human neutrophil granulocytes. Myeloperoxidase, which has a physiologic role in antibacterial defence, and antioxidant steroids, like estradiol and hydrocortisone have a variably positive effect on bactericidal activity. Although the donors were both males and females, we did not find sex-related differences in the bacterial killing of PMNs.

The results of our *in vivo* studies are also in accordance with these. The behaviour of the animals, the death rates, development of ascites, and most importantly the reisolation of bacteria all support the negative impact of indomethacin on the course of the *P. multocida* infection. Laboratory findings also show decreased coping capability of infected and indomethacin treated rats: higher white blood cell counts, increased neutrophil ratio, anaemia, decreased concentration of serum proteins (albumin, globulin, total protein) and CRP. Serum proteins, being mediators of the humoral immune system, give the base of the inflammatory response against microorganisms. Serum albumin is decreased in inflammatory diseases due to reduced hepatic synthesis [18]. CRP is an acute phase protein, that can influence multiple stages of inflammation and has both proinflammatory and antiinflammatory effect [19,20]. The collective decrease of these parameters implies a deteriorated bactericidal capacity. It is interesting to note, that indomethacin tends to lower serum albumin, even without infection. As 90-99% of indomethacin is bound to albumin in the bloodstream, reduced albumin levels lead to an increased concentration of free indomethacin, and thus an even worse antibacterial activity during infection [21].

As expected, castration led to weak clinical state, however, in the untreated groups, castration did not worsen reisolation and laboratory parameters after infection with *P. multocida*. On the other hand, testosterone treatment of castrated animals seems to cause anaemia and weaker bactericidal capacity when compared to non-castrated and testosterone-treated rats, but better WBC and CRP results when compared to the castrated, untreated group. Testosterone treatment of non-castrated animals resulted in stronger bactericidal effect (less reisolated bacteria) and lower WBC count after infection compared to the non-castrated, untreated group. Taken together, testosterone seems to improve the levels of inflammatory parameters compared to the respective control.

The effect of testosterone on the immune system has been studied by others with various results. It has been shown that testosterone leads to delayed acquired immunity by directly affecting T or B cells [22], reducing NK cell activity [23], limiting lymphocyte proliferation, and decreasing immunoglobulin and cytokine production [24-26]. Testosterone has been shown to impair TNF and NO production by macrophages, and to suppress NF-κB signal transduction, which has a substantial role in mediating proinflammatory cytokine production. It can also enhance the production of anti-inflammatory cytokines like IL-10 and IL-4 [23]. However, in another study elevated proinflammatory cytokines such as TNF-α, IL-6 and IL-1-ß were found in male rats compared to females [27].

Estradiol treatment of castrated rats seems to enhance bactericidal defence mechanisms most, as reisolation of *P. multocida* showed the lowest colony numbers, and decreased CRP and WBC count could mean that the infection was less severe due to estradiol pre-treatment. E_2_ also led to lower platelet count, thus decreasing the risk of thromboembolism in these animals.

In the literature E_2_ has been shown to have a dual effect according to its concentration. Low doses of E_2_ enhance the production of proinflammatory cytokines (IL-1, IL-6, TNF- α) and T helper cell type 1 (Th1) responses, while high or sustained E_2_ concentrations are associated with an increased susceptibility to infections [28], reduced proinflammatory cytokine production and enhanced Th2 responses and humoral immunity [23,29]. This effect is thought to be mediated by estrogen receptor signalling, which suppresses NF-κB activity [30]. E_2_ can inhibit the proliferation of mononuclear leukocytes and reduce NK cell cytotoxicity [26]. In a study with steers, administration of high doses of E_2_ did not affect total and differential WBC count, lymphocyte blastogenesis and neutrophil function [31]. When given to castrated mice, E_2_ did not exhibit immunosuppressive effects [22], but another study concluded that intracellular bacterial killing in PMNs is impaired by physiological levels of E_2_, probably due to a decrease in MPO activity [32,33].

According to our previous results, antioxidant steroids increase MPO activity, which leads to an elevation of the concentration of hypochlorite, the end product of MPO induced peroxidation [14-16]. This inhibits the production of superoxide anion through a negative feedback effect on NOX, which ultimately leads to the decrease of free radicals derived from superoxide, including hypochlorite [14]. Thus the negative feedback inhibition of NOX is resolved, and this again results in increased superoxide generation. Indomethacin treatment, on the other hand, acts the opposite way [14]. The effect of MPO and indomethacin in the actual setup probably depends greatly on the duration of treatment. In the present setting, the two-hour treatment with indomethacin did not cause significant changes, while MPO increased bactericidal activity. The five-day indomethacin treatment of rats diminished bactericidal activity, probably by decreasing hypochlorite anion concentration. According to our hypothesis mentioned above, this might lead to enhanced superoxide anion levels through the inhibition of the negative feedback and thus bactericidal activity would increase as well. However, this potential regulating mechanism could not reach the level of increased bactericidal activity, because the diminished hypochlorite concentration in the early phase determined the prognosis of the infection.

Antioxidant steroid hormones seem to improve bactericidal activity in the short run. In the long run, however, higher hypochlorite levels produced by increased MPO activity may exhibit negative feedback on NOX, leading to decreased superoxide generation and bactericidal activity. Impaired immunity is a known consequence of chronic steroid overproduction or treatment [34].

It is important to note that our results in the two experimental models are difficult to compare, one being an *ex vivo*, the other an *in vivo* setup. As detailed above, sex steroids play a role in a number of innate and acquired immune functions and a variety of immune cell types have sex steroid receptors [23,24,26,28]. The improved bactericidal activity of PMNs by estradiol confirmed by our present experiment is just one of these effects, and in vivo more complex processes are modulated by sex steroids.

The low number of samples in both the *ex vivo* and *in vivo* experiments is a limitation of our study, so our findings need further confirmation with greater sample numbers in the future.

## Conclusions

In our experimental models MPO, estradiol, and hydrocortisone improve bacterial killing activity of PMNs. Indomethacin treatment and castration weaken immune responses and the clinical state of infected rats. These experiments did not confirm the supposed beneficial effect of indomethacin in the treatment of septic patients. On the other hand, testosterone, and even more so estradiol had a beneficial effect in bacterial infection. This finding needs further confirmation with greater sample sizes, but based on our results, it is possible, that short term adjuvant steroid treatment might have a beneficial effect in the treatment of bacterial infections and sepsis. In order to avoid side effects of steroid hormone treatment, it would be reasonable to design an endocrinologically inactive synthetic compound with the antioxidant features of the analysed steroids.

## Competing interests

This study was funded by Gedeon Richter Plc.

## Authors’ contributions

JS participated in the writing of the manuscript, planning of the study, collection of the data and the analysis of results. ZV carried out animal treatments and bacteria reisolations and participated in the writing of the manuscript. AG carried out the ex vivo experiments and participated in the writing of the manuscript. PV carried out laboratory measurements in the in vivo experiments and participated in the writing of the manuscript. DS carried out the anaesthesia and section of rats. IM participated in the design of the study and the collection of data. SV participated in the design of the study and the analysis of results. ED participated in the design of the study and performed the statistical analysis. TM participated in the animal treatments, supervised the study and gave institutional support. ZT supervised the study and gave institutional support. BS supervised the study and participated in the analysis of results. KN participated in the ex vivo experiments, supervised the study and gave institutional support. KR supervised the study and gave institutional support. GB planned study concept and design, analyzed and interpreted data and revised the manuscript. All authors read and approved the final manuscript.
